# Man With Dyspnea and Chest Pain

**DOI:** 10.1016/j.acepjo.2025.100293

**Published:** 2025-12-05

**Authors:** Chia-Ching Chen

**Affiliations:** Department of Emergency Medicine, Chang Bing Show-Chwan Memorial Hospital, Changhua, Taiwan, Republic of China

**Keywords:** POCUS, pneumopericardium, lung neoplasms

## 1 Case Report

**Figure 1:**
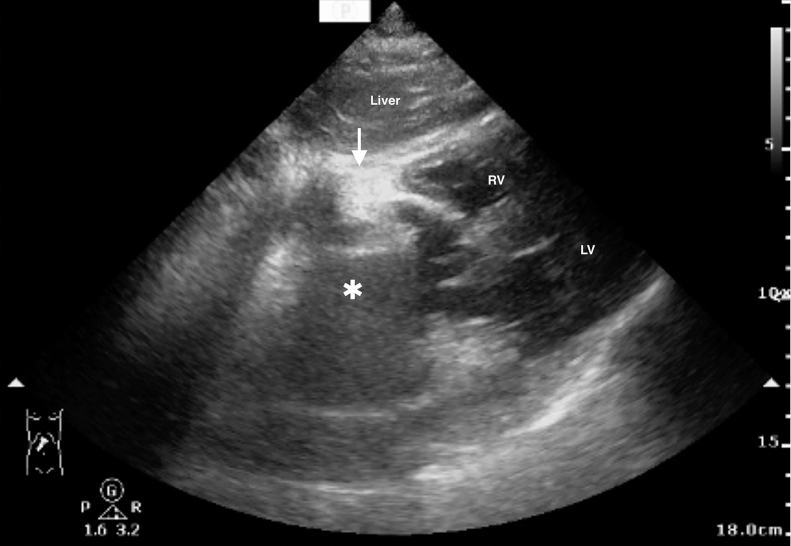
Point-of-care ultrasonography with subcostal four-chamber view demonstrates the liver (superior), left ventricle (LV), and right ventricle (RV). Multiple hyperechoic foci (arrow) with posterior dirty shadowing artifacts (asterisk) fill the pericardial space, consistent with pneumopericardium. The right atrium is completely obscured by the air artifacts.

**Figure 2:**
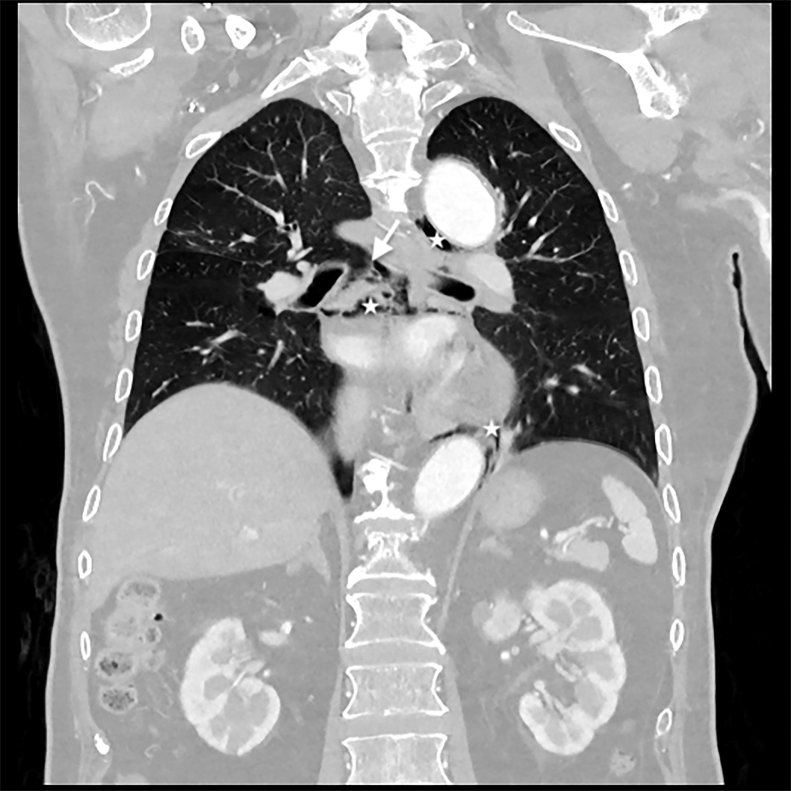
Coronal reformatted contrast-enhanced chest computed tomography demonstrates tracheomediastinal fistula (arrow) at the level of the carina with extensive pneumomediastinum and pneumopericardium (star) secondary to left upper lobe lung malignancy.

A 71-year-old man with lung cancer presented to the emergency department with 12 hours of dyspnea and chest pain. Vital signs showed a blood pressure of 139/99 mm Hg, a heart rate of 148 beats per min, a respiratory rate of 28 breaths/min, temperature 38.4° C (101.12° F), and oxygen saturation 88% on room air. Physical examination revealed a crunching sound synchronous with the heartbeat (Hamman’s sign). Point-of-care ultrasonography (POCUS) (Fig 1 and Video 1) demonstrated multiple swirling hyperechoic foci within the pericardial space. Computed tomography (Fig 2) confirmed pneumomediastinum and pneumopericardium with tracheomediastinal fistula at the proximal right main bronchus.

**Video 1:**
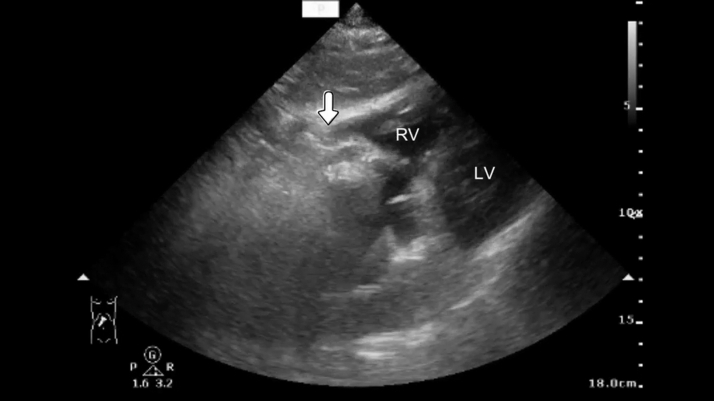
Real-time point-of-care ultrasonography with subcostal four-chamber view and inferior vena cava long-axis view demonstrates multiple hyperechoic foci with posterior dirty shadowing artifacts swirling within the pericardial space, consistent with pneumopericardium. The characteristic movement of air bubbles with cardiac motion is clearly visible.

## 2 Diagnosis: Pneumopericardium and Pneumomediastinum Secondary to Lung Cancer

Pneumopericardium, air within the pericardial sac, is rare and life-threatening. In malignancy, it results from tumor invasion, causing airway-mediastinal fistula formation.^1^ Hamman’s sign is a classic finding. POCUS is increasingly used in emergency departments to rapidly identify cardiac pathology,^2^ including pneumopericardium, which appears as hyperechoic foci with reverberation artifacts. Swirling echoes represent air bubbles moving with cardiac motion. Management includes high-flow oxygen, analgesia, and antibiotics. Tension pneumopericardium requires urgent pericardiocentesis or surgical decompression. The patient was admitted for supportive care and palliative management.

## Funding and Support

By *JACEP* Open policy, all authors are required to disclose any and all commercial, financial, and other relationships in any way related to the subject of this article as per ICMJE conflict of interest guidelines (see www.icmje.org). The authors have stated that no such relationships exist.

## Conflict of Interest

All authors have affirmed they have no conflicts of interest to declare.
